# Diabetes Mellitus and Clinical Outcomes in Carotid Artery Revascularization Using Second-Generation, MicroNet-Covered Stents: Analysis from the PARADIGM Study

**DOI:** 10.1155/2022/8691842

**Published:** 2022-09-26

**Authors:** Adam Mazurek, Anna Borratynska, Urszula Gancarczyk, Lukasz Czyz, Martyna Sikorska, Lukasz Tekieli, Bartosz Sobien, Marcin Jakiel, Mariusz Trystula, Tomasz Drazkiewicz, Piotr Podolec, Piotr Musialek

**Affiliations:** ^1^Jagiellonian University, Department of Cardiac & Vascular Diseases, John Paul II Hospital, Krakow, Poland; ^2^John Paul II Hospital, Neurology Outpatient Department, Krakow, Poland; ^3^Jagiellonian University, Department of Interventional Cardiology, John Paul II Hospital, Krakow, Poland; ^4^John Paul II Hospital, Department of Vascular Surgery, Krakow, Poland

## Abstract

**Introduction:**

Carotid artery stenting (CAS) using conventional (single-layer) stents is associated with worse clinical outcomes in diabetes mellitus (DM) vs. non-DM patients: an effect driven largely by lesion-related adverse events. CAS outcomes with MicroNet-covered stents (MCS) in diabetic patients have not been evaluated.

**Aim:**

To compare short- and long-term clinical outcomes and restenosis rate in DM vs. non-DM patients with carotid stenosis treated using MCS.

**Materials and Methods:**

In a prospective study in all-comer symptomatic and increased-stroke-risk asymptomatic carotid stenosis, 101 consecutive patients (age 51-86 years, 41% diabetics) underwent 106 MCS-CAS. Clinical outcomes and duplex ultrasound velocities were assessed periprocedurally and at 30 days/12 months.

**Results:**

Baseline characteristics of DM vs. non-DM patients were similar except for a higher prevalence of recent cerebral symptoms in DM. Type 1 and type 1+2 plaques were more prevalent in DM patients (26.7% vs. 9.8%, *p* = 0.02; 62.2% vs. 37.7%, *p* = 0.01). Proximal embolic protection was more prevalent in DM (60% vs. 36%; *p* = 0.015). 30-day clinical complications were limited to a single periprocedural minor stroke in DM (2.4% vs. 0%, *p* = 0.22). 12-month in-stent velocities and clinical outcomes were not different (death rate 4.8% vs. 3.3%; *p* = 0.69; no new strokes). Restenosis rate was not different (0% vs. 1.7%, *p* = 0.22).

**Conclusions:**

MCS may offset the adverse impact of DM on periprocedural, 30-day, and 12-month clinical complications of CAS and minimize the risk of in-stent restenosis. In this increased-stroke-risk cohort, adverse event rate was low both in DM and non-DM. Further larger-scale clinical datasets including extended follow-ups are warranted.

## 1. Introduction

Diabetes mellitus type 2 (DM) not only significantly increases the risk of atherosclerotic cardiovascular disease but it is also associated with worse clinical outcomes in coronary artery disease and peripheral artery disease interventions, including interventional management of carotid artery stenosis [1]. With conventional carotid artery stenting (CAS), DM patients more frequently experience complications including peri- and postprocedural strokes [2]. Because of worse outcomes of conventional (single layer, also termed “first-generation” [3]) carotid stents in diabetic patients [2], DM has been considered, in some centers, a relative contraindication to using the endovascular route of carotid revascularization.

Regarding longer-term outcomes of atherosclerotic lesions treated with stents, DM is a major risk factor for in-stent restenosis both in the coronary tree and in the carotid arteries [4–6]. Recent evidence indicates that, in the coronaries, the adverse impact of diabetes on postprocedural complications may be offset by using new-generation drug-eluting stents [7]. In CAS, MicroNet-covered stents (MCS) have been recently shown to effectively prevent periprocedural and long-term lesion-related cerebral embolism [8], and they may reduce clinical complications of CAS [9, 10], presenting a major improvement in the endovascular armentarium [11]. MCS outcomes in the diabetic population have not been evaluated.

We hypothesized that the dual-layer MicroNet-covered carotid stent use in CAS in primary and secondary stroke prevention in diabetic patients might offset—by plaque sealing with inhibition of cerebral embolism and the stented lumen optimization [8, 12]—the impact of DM on adverse clinical events and restenosis rate by 12 months.

## 2. Materials and Methods

PARADIGM (Prospective evaluation of All-comer peRcutaneous cArotiD revascularisation in symptomatic and Increased-risk asymptomatic carotid artery stenosis using CGuard™ MicroNet-covered embolic prevention stent system) is a prospective academic study in all-referral-tracked symptomatic and asymptomatic carotid stenosis, with a multispecialty neurovascular team (angiologist/cardiologist, vascular surgeon, neurologist) [13] decision-making on revascularization. Acute clinical presentation was defined as presentation within 14 days from symptoms of ipsilateral cerebral ischemia. The study enrolled unselected, consecutive patients with an independent neurologic evaluation at baseline, periprocedurally and at 1 and 12 months, and with event adjudication by an independent Clinical Events Committee (CEC) [13, 14]. CEC consisted of neurologist, cardiologist, and a vascular medicine specialist. Duplex ultrasound (DUS) was performed preprocedurally (lesions characteristics and velocities) and at 30 days and 12 months postprocedurally (in-stent material, velocities). Carotid plaque type [15] was routinely assessed on preprocedural DUS by consensus of two DUS analysts not performing interventions. Distal (filters) or proximal cerebral protection (transient flow reversal) was used in CAS [13]. The dual-layered study stent is 6F-compatible; thus, 6F sheaths were routinely used in case of filter-protected procedures. In proximal-protected cases, MoMa 9F or FlowGate 8F balloon catheters were typically used to ensure effective flow reversal to protect the brain against embolism. Stents were routinely “coronary-like” optimized, using appropriately sized balloons and high pressures [13]. The threshold for suspected restenosis on DUS follow-up was peak-systolic velocity of at least 175 cm/s [16]. Suspicion of in-stent restenosis (ISR) triggered invasive angiographic verification [12].

The PARADIGM study was registered with local Ethics Committee and has been conducted in accordance with the Declaration of Helsinki (1964). All study participants gave written consent form. The study record is registered and maintained at http://Clinicaltrial.gov (NCT04271033).

### 2.1. Study Outcomes

Clinical outcomes of interest included death, stroke, and myocardial infarction (MI), assessed periprocedurally, at 30 days and at 12 months). MI and stroke definitions were according to guidelines. The fundamental imaging-based outcome clinical relevance was the rate of ISR.

### 2.2. Study Data External Monitoring

Study data monitoring (100%) was performed, through an academic research grant, by an external clinical research organization (CRO).

### 2.3. Statistical Analysis

Continuous variables were compared using Student's *t*-test. Differences in proportions were evaluated with the chi-square test. The level of statistical significance was determined at *p* < 0.05. All numerical data were presented as mean and standard deviation, median and range, or as proportions. Statistical calculations were performed using STATISTICA data analysis software, version 10.0 (StatSoft, Inc., Tulsa, OK, USA).

## 3. Results

Baseline characteristics of DM vs. non-DM patients and lesions were similar and are depicted in Tables 1 and 2. Clinical presentation with recent symptoms (≤14 days) occurred more frequently in DM patients (20% vs. 2%; *p* = 0.003; data for DM vs. non-DM cohort). Increased-risk carotid plaque type (type 1 and type 1+2 according to Gray-Weale classification [15]) was more prevalent in DM patients (26.7% vs. 9.8%, *p* = 0.02 and 62.2% vs. 37.7%, *p* = 0.01). Combined prevalence of soft, highly-lipid carotid plaques on the one end of the spectrum, and highly calcific on the other [17], was also greater in the DM patients (82.2% % vs. 50.8%, *p* < 0.001). Consistent with the “tailored CAS” algorithm [18], the use of proximal embolic protection systems was significantly more frequent in DM patients (60.0% vs. 36.1%; *p* = 0.015).

In both study cohorts (i.e., DM and non-DM patients), most lesions were predilated (Table 2). There were no differences between the study cohorts in primary stenting rate (6.7% vs. 9.8% *p* = 0.56), mean stent diameters (8.13 mm vs. 8.21 mm, *p* = 0.57; range 7-9 mm for both), and stent length (32.2 mm vs. 33.6 mm, *p* = 0.25, range 30-40 mm in both groups). Postdilation balloon catheter diameters and pressures were also similar (maximal pressure 20.22 atm. vs. 19.31 atm, *p* = 0.18; balloon catheter diameter 5.12 mm vs. 5.24 mm, *p* = 0.09). Vascular closure device use was similar (53% vs. 57%, *p* = 0.68).

Periprocedural and 30-day outcomes were not different. There was a single periprocedural minor stroke (2.4% vs. 0%, *p* = 0.22) but no major stroke, no death, and no MI occurred. Access site closure device use rate was 55.7%, with no significant difference between DM and non-DM patients (53% vs. 57%, *p* = 0.68). The rate of access site complications requiring any intervention (such as false aneurysm thrombin injection or surgery) was 4.7% (4.4% vs. 4.9%, *p* = 0.91).

By 30 days, there were no new strokes, no postprocedural MIs, and no deaths. 12-month clinical outcomes were similar in both groups (*p* = 0.70), including 4.8% deaths in the DM cohort (mechanisms: urosepsis, chronic heart failure exacerbation) vs. 3.3% in non-DM (pulmonary embolism, pulmonary cancer). No new strokes or MIs occurred by 12 months in either group. Furthermore, restenosis rate was not different (0% vs. 1.7%, *p* = 0.22). The single in-stent restenosis (0.94%) was effectively treated at 13 months with a drug-eluting balloon under IVUS control and distal embolic protection system use, and there was no relapse. At 12 months, a slight (though statistically significant) increase in in-stent velocities was noted in non-DM patients (peak-systolic velocity at 30 days 0.64 ± 0.24 m/s vs. 0.78 ± 0.31 m/s at 12 months; *p* = 0.007; end-diastolic velocity at 30 days 0.17 ± 0.07 m/s vs. 0.21 ± 0.09 m/s at 12 months; *p* = 0.006) but, interestingly, this did not reach statistical significance in DM patients (peak-systolic velocity at 30 days 0.73 ± 0.34 m/s vs. 0.87 ± 0.59 m/s at 12 months; *p* = 0.16; end-diastolic velocity at 30 days 0.18 ± 0.08 m/s vs. 0.22 ± 0.18 m/s at 12 months; *p* = 0.14). At each DUS time point, however, no significant differences occurred between the DM and non-DM patients (Tables 2 and 3).

## 4. Discussion

Our present analysis indicates that the MicroNet-covered stent use in CAS in diabetic patients (1) is associated with a low periprocedural complication rate in this otherwise high-risk population [19, 20] similar to that seen with this novel stent type in general/non-DM populations [9, 21, 22]; (2) there is absence of any signal of increased in-stent restenosis rate in MicroNet-covered stent implants in diabetic patients, and (3) the rate of 12-month adverse clinical events in DM patients treated with the use of MicroNet-covered stents is similar to that in non-DM patients.

Diabetes, independent from other risk factors, confers ≈2-fold excess risk for a wide range of atherosclerotic vascular diseases [1, 5]. Recent analysis of data for nearly 700 000 patients from 102 prospective studies showed, with diabetes, adjusted hazard ratio for ischemic stroke of 2.27 (95% CI 1.95-2.65) [5]. Women with diabetes may be at a particularly large risk [23]. In atherosclerotic carotid stenosis, luminal narrowing > 50% and diabetes duration significantly increase the risk of stroke (*p* < 0.001) [6]. This risk is not amenable to any sufficient control with classic optimized medical therapy (high-dose statin titrated to achieve guideline-indicated LDL-cholesterol level, antiplatelet agent, ACEI/ARB) [13], and it is also not reduced with gliflozines [24]. Thus, procedural low-risk carotid revascularization with plaque sealing [11] (on top of maximized medical therapy) might play an important role in durable stroke risk reduction [25] in diabetic patients with increased stroke-risk carotid stenosis. An important aspect of diabetes in the context of conventional cardiovascular interventions is that DM is associated with a permanent prothrombotic state that may importantly enhance the risk of stent thrombosis and restenosis [26]. The present study indicates that endovascular carotid revascularization using MCS is safe and effective also in diabetic patients who, with prior-generation CAS, are at increased risk of adverse events. This work expands our prior findings for patients with highly calcific carotid lesions [27] (a “classic” contraindication to conventional-stent CAS, similar to DM presenting, in view of some operators, a contraindication to conventional CAS), showing that CAS using a second-generation, MicroNet-covered stent can be performed safely and effectively also in increased-risk populations.

Our present work provides grounds for a hypothesis that the risk of lesion-related adverse events in CAS might be, at least in part, offset using MCS—similar to the role played by drug-eluting (rather than bare-metal) stents in coronary revascularization in diabetic patients [7, 28, 29]. This hypothesis requires further elucidation in larger patient cohorts.

First-generation (single-layer) carotid stents fail to sequestrate the atherosclerotic plaque [3, 30–32]. The unwanted phenomenon of plaque prolapse is not eliminated using closed-cell single-layer stents [31, 32]. In contrast, it can be effectively abolished with MCS use [11, 12]. This may be clinically important as a large proportion of peri- and postprocedural CAS complications is lesion-level based [11]. There is ample evidence that in CAS using conventional carotid stents, diabetes significantly increases the risk major adverse events including perioperative stroke (OR 1.38, 95% CI: 1.02-1.88, *p* = 0.04), death (OR 1.94, 95% CI: 1.36-2.75, *p* = 0.0002), the composite endpoint of perioperative stroke or death (OR 1.80, 95% CI: 1.32-2.47, *p* = 0.0002), and the risk of long-term death (OR 1.57, 95% CI: 1.22-2.03, *p* = 0.0005) [2]. DM patients with carotid disease suffer more frequently from symptoms of cerebral ischemia, with DM-associated increased thrombotic activity as a leading potential mechanism [33].

In absence of plaque sequestration [11], diabetes significantly increases the risk of in-stent restenosis [34]. In conventional carotid stents, this mid/long-term complication may not be clinically benign [35], and it importantly contributes to CAS overall adverse events in relation to CEA [36]. Recent data suggest that a proportion of “in-stent restenoses” in single-layer (conventional) stents may represent plaque progression into the lumen [12]; an adverse phenomenon is amenable to elimination with MCS [11, 12]. This is important because “in-stent restenosis” is associated with an increased risk of recurrent stroke [24, 25], and it poses a significant management challenge [12, 37, 38].

Recent prospective analysis of conventional carotid stent CAS in 563 symptomatic patients (17.6% with DM) demonstrated a significant increase in the risk of ipsilateral stroke with diabetes (HR = 2.361; 95% CI: 1.052–5.302, *p* = 0.037, at 30 days) [19]. The single-layer stents used in that study included mainly Carotid Wallstent (77.9%) and Precise (6.7%) [19]. By 4 years, diabetes increased the risk of ipisilateral stroke by 69.3%, increased the risk of stroke or vascular death (HR = 2.091; 95% CI: 1.267–3.451, *p* = 0.004), and the risk of stroke or any death (HR = 1.921; 95% CI: 1.269–2.908, *p* = 0.002) [19]. Moreover, that study clearly demonstrated that ^“^ISR^”^ > 50% increased ipsilateral stroke during follow-up by >2-fold (HR = 2.187; 95% CI: 1.173–4.078, *p* = 0.014), a finding particularly important in view of the recent data that “ISR” in single-layer stents may represent in-stent atherosclerotic plaque progression [12]. Similarly, analysis of 946 consecutive (38.2% diabetic patients) [20] first-generation-stent CAS identified diabetes as a leading risk factor for in-stent restenosis (OR = 2.82; 95% CI: 1.13–7.15, *p* = 0.025). Single-layer carotid stents in that study included Carotid Wallstent (64.6%), Acculink (14.8%), Xact (10.4%), Vivex (6.0%), Protegè (3.6%), Precise (1.4%), and Cristallo (0.3%) [20]. Normal in-stent velocities in MCS at 30 days and 12 months, similar in non-DM and DM patients (Table 2), reflect the device normal healing and are consistent with a low restenosis rate in MCS reported by other investigators [9, 22].

Our present work needs to be viewed in the context of other pivotal studies using “mesh-covered” stents, particularly those including second-generation stents other than the MCS [39–41]. In recent dual-metallic layer stent studies by Nerla and colleagues [39, 40], their 150 CAS patient population included 27% diabetics. In absence of CAS peri- and postprocedural events by 30 days, there were three deaths and three restenosis by 12 months [39, 40]. It is unclear, however, whether the affected patients were diabetic or nondiabetic, and evaluation of any potential impact of DM on ISR in dual-metallic layer stents is warranted. Regarding MCS, two large-scale Italian registries have been recently published: IRONGUARD-1 [42, 43] and IRONGUARD-2 [10, 44]. The IRONGUARD-1 study population of 200 patients included 28% diabetics. In 61 patients, postprocedural DW-MRI was performed, indicating no difference in new cerebral ischemic lesions between DM and non-DM patients. For the five periprocedural minor stroke patients, their DM status was unfortunately not given. Nevertheless, 12-month data showed no new ischemic strokes (suggesting lack of any diabetes impact on the postprocedural stroke rate) and a single ISR that required treatment. The IRONGUARD-2 study population (733 consecutive patents) had a larger proportion of DM patients (36%). Peri- and postprocedural adverse clinical outcomes (cumulative 30-day death/stroke/MI rate of 1.2%) showed no difference between DM and non-DM patients [10]; this corroborates the present findings in our study. Also 12-month data IRONGUARD-2 showed no difference in death/stroke/MI between DM vs. non-DM patients. There were 6 incidences of ISR; however, no DM status was provided for these patients [44]. Overall, the IRONGUARD-1 [42, 43] and IRONGUARD-2 [10, 44] clinical results are in concordance with our present findings. Long-term outcomes of dual-metallic layer stents in particular require further evaluation with respect to a potential role of DM [22].

Recent evidence shows that, in single-layer carotid stent CAS, plaque characteristics play a significant role in “ISR” [45, 46]. This is consistent with in-stent plaque progression as a clinically relevant mechanism of in-stent restenosis [12]; a phenomenon eliminated by plaque sealing [11] that may play a particularly important role in diabetic patients is known to be at increased risk of “ISR.”

### 4.1. Limitations

This is an exploratory posthoc analysis from a single-center study, and further larger-scale prospective data are needed to corroborate our findings. Moreover, our present analysis is limited to transfemoral CAS using the MicroNet-covered stent. MCS may play an important role in reducing peri- and postprocedural events also in transcervical carotid revascularization [47]. With the use of a classic first-generation carotid stent in trans-cervical carotid revascularization under “dynamic” flow reversal, in a population including 37.4% diabetic patients, periprocedural stroke/death rate was increased 2-fold (odds ratio 1.99; 95% CI: 1.01–3.92; *p* = 0.046) in symptomatic vs. asymptomatic patients, and the restenosis rate remained high at ≈4% in ≈12months [48].

## 5. Conclusions

MicroNet-covered stent use may offset the adverse impact of DM on periprocedural, 30-day, and 12-month clinical complications of CAS as well as the adverse impact of DM on in-stent restenosis seen with conventional carotid stents in diabetic patients. In this increased stroke-risk cohort, 12-month death/stroke/myocardial infarction and restenosis risk was low both in DM and non-DM patients. Further, larger-scale multicentric clinical data are needed.

## Figures and Tables

**Table 1:** Clinical characteristics of the study cohorts.

	DM (*n* = 41)	Non-DM (*n* = 60)	*p* value
Male (*n*, %)	30 (73.2)	41 (68.3)	0.60
Mean age ± SD (years, min-max)	70.54 ± 7.56 (51-86)	67.83 ± 7.23 (53-81)	0.08
Symptomatic (*n*, %)	25 (61)	30 (50)	0.27
Acute presentation (symptoms < 14 days) (n, %)	8 (19.5)	1 (1.7)	0.003
Prior stroke (*n*, %)	20 (48.8)	21 (35)	0.10
RTh (*n*, %)	1 (2.4)	5 (8.3)	0.22
AF (*n*, %)	4 (9.8)	5 (8.3)	0.81
Contralateral CAS/CEA (*n*, %)	2 (4.9)	10 (16.7)	0.07
CAD (*n*, %)	28 (68.3)	36 (60)	0.40
MI (*n*, %)	17 (41.5)	15 (25)	0.08
h/o CABG or PCI (*n*, %)	19 (46.3)	21 (35)	0.25
Arterial hypertension (*n*, %)	36 (87.8)	54 (90)	0.72

RTh: radiotherapy; AF: atrial fibrillation; CAS: carotid artery stenting; CEA: carotid endarterectomy; CAD: coronary artery disease; CABG: coronary artery bypass grafting; PCI: percutaneous coronary intervention; h/o: history of.

**Table 2:** Study lesions and index procedure characteristics and in-stent velocities at follow-up.

	DM (*n* = 45)	Non-DM (*n* = 61)	*p* value
RICA (*n*, %)	25 (55.6)	32 (52.5)	0.75
Both sides (*n*, %)	4 (8.9)	1 (1.6)	0.08
Diameter stenosis; QCA (%)	84.1 ± 0.09	82.2 ± 0.10	0.34
Lesion length (mm)	18.7 ± 6.28	20.4 ± 5.60	0.15
Plaque type			
Type 1 (%)^∗^	12 (26.7)	6 (9.8)	0.02
Type 2 (%)^∗^	16 (35.6)	17 (27.9)	0.40
Type 3 (%)^∗^	4 (8.9)	16 (26.2)	0.02
Type 4 (%)^∗^	4 (8.9)	14 (22.9)	0.06
Type 5 (%)^∗^	9 (20.0)	8 (13.1)	0.34
Type 1+2 (%)^∗^	28 (62.2)	23 (37.7)	0.01
Type 1+2+5 (%)^∗^	37 (82.2)	31 (50.8)	<0.001
Baseline PSV (m/s)	3.64 ± 1.06	3.75 ± 1.3	0.65
Baseline EDV (m/s)	1.21 ± 0.59	1.28 ± 0.72	0.58
Proximal EPD (*n*, %)	27 (60)	22 (36.1)	0.014
Distal EPD (*n*, %)	18 (40)	39 (63.9)	
Direct stenting (*n*, %)	3 (6.7)	6 (9.8)	0.56
Max. postdilatation pressure (mean ± SD; atm)	20.22 ± 3.44	19.31 ± 4.44	0.18
Postdilatation balloon catheter diameter (mean ± SD; mm)	5.12 ± 0.34	5.24 ± 0.34	0.09
Residual diameter stenosis; QCA (%)	6.1 ± 0.08	5.9 ± 0.05	0.84
Stent diameter (mean ± SD; mm)	8.13 ± 0.73	8.21 ± 0.69	0.57
Stent length (mean ± SD; mm)	32.22 ± 4.2	33.61 ± 7.97	0.25
Vascular closure device use (*n*, %)	24 (53)	35 (57)	0.68
30-day follow-up			
PSV (m/s ± SD)	0.73 ± 0.34	0.64 ± 0.24	0.17
EDV (m/s ± SD)	0.18 ± 0.08	0.17 ± 0.07	0.60
12-month follow-up			
PSV (m/s ± SD)	0.87 ± 0.59	0.78 ± 0.31	0.37
EDV (m/s ± SD)	0.22 ± 0.18	0.21 ± 0.09	0.74

RICA: right internal carotid artery; QCA: quantitative comparative analysis; PSV: peak systolic velocity; EDV: end diastolic velocity; EPD: embolic protection device. Note that continuous variables presented as mean ± SD; ^∗^DUS plaque type according to Gray-Weale classification (type 1: uniformly unechoic or hypoechoic; type 2: predominantly (>50%) hypoechoic; type 3: predominantly (>50%) hyperechoic; type 4: uniformly hyperechoic; type 5: uniformly echogenic with posterior shadowing (calcified plaque)) [14].

**Table 3:** In-stent velocity change in diabetes and non-diabetes patents.

30 days–12 months	*p* value
*Δ* PSV in DM	0.16
*Δ* PSV in non-DM	0.007
*Δ* EDV in DM	0.14
*Δ* EDV in non-DM	0.006

PSV: peak systolic velocity; EDV: end diastolic velocity for raw velocity values see Table 2.

## Data Availability

Inquiries for the study data can be directed to the corresponding author.

## References

[1] Wei L. M., Zhu Y. Q., Bao Y. Q. (2019). Atherosclerosis in intracranial or extracranial vessels in diabetic patients and the association with stroke subtype. *Quantitative Imaging in Medicine and Surgery*.

[2] Hussain M. A., Bin-Ayeed S. A., Saeed O. Q., Verma S., Al-Omran M. (2016). Impact of diabetes on carotid artery revascularization. *Journal of Vascular Surgery*.

[3] Nakagawa I., Kotsugi M., Park H. S. (2021). Near-infrared spectroscopy carotid plaque characteristics and cerebral embolism in carotid artery stenting. *EuroIntervention*.

[4] Hong S. J., Kim M. H., Ahn T. H. (2006). Multiple predictors of coronary restenosis after drug-eluting stent implantation in patients with diabetes. *Heart*.

[5] Sarwar N., Gao P., Seshasai S. R. (2010). Diabetes mellitus, fasting blood glucose concentration, and risk of vascular disease: a collaborative meta-analysis of 102 prospective studies. *The Lancet*.

[6] Noh M., Kwon H., Jung C. H. (2017). Impact of diabetes duration and degree of carotid artery stenosis on major adverse cardiovascular events: a single-center, retrospective, observational cohort study. *Cardiovascular Diabetology*.

[7] Paszek E., Zajdel W., Musiałek P. (2019). Percutaneous management of long and diffused coronary lesions using newer generation drug-eluting stents in routine clinical practice: long-term outcomes and complication predictors. *Polskie Archiwum Medycyny Wewnętrznej= Polish Archives of Internal Medicine*.

[8] Karpenko A., Bugurov S., Ignatenko P. (2021). Randomized controlled trial of conventional versus MicroNet-covered stent in carotid artery revascularization. *JACC. Cardiovascular Interventions*.

[9] Stabile E., de Donato G., Musialek P. (2018). Use of dual-layered stents in endovascular treatment of extracranial stenosis of the internal carotid artery: results of a patient-based meta-analysis of 4 clinical studies. *JACC. Cardiovascular Interventions*.

[10] Sirignano P., Stabile E., Mansour W. (2020). 1-month results from a prospective experience on CAS using CGuard stent system: the IRONGUARD 2 study. *JACC. Cardiovascular Interventions*.

[11] Musialek P., Roubin G. S. (2019). Commentary: Double-layer carotid stents: from the clinical need, through a stent-in-stent strategy, to effective plaque isolation… the journey toward safe carotid revascularization using the endovascular route. *Journal of Endovascular Therapy*.

[12] Tekieli L., Mazurek A., Pieniazek P., Musialek P. (2022). Symptomatic atherosclerotic plaque progression in a first-generation carotid stent: management and 5-year clinical and imaging outcome-a case report. *European Heart Journal-Case Reports*.

[13] Musialek P., Mazurek A., Trystula M. (2016). Novel PARADIGM in carotid revascularisation: Prospective evaluation of All-comer peRcutaneous cArotiD revascularisation in symptomatic and Increased-risk asymptomatic carotid artery stenosis using CGuard™ MicroNet-covered embolic prevention stent system. *EuroIntervention*.

[14] Mazurek A., Borratynska A., Malinowski K. P. (2020). MicroNET-covered stents for embolic prevention in patients undergoing carotid revascularisation: twelve-month outcomes from the PARADIGM study. *EuroIntervention*.

[15] Gray-Weale A. C., Graham J. C., Burnett J. R., Byrne K., Lusby R. J. (1988). Carotid artery atheroma: comparison of preoperative B-mode ultrasound appearance with carotid endarterectomy specimen pathology. *The Journal of Cardiovascular Surgery*.

[16] Setacci C., Chisci E., Setacci F., Iacoponi F., de Donato G. (2008). Grading carotid intrastent restenosis: a 6-year follow-up study. *Stroke*.

[17] Paprottka K. J., Saam D., Rübenthaler J. (2017). Prevalence and distribution of calcified nodules in carotid arteries in correlation with clinical symptoms. *La Radiologia Medica*.

[18] Pieniazek P., Musialek P., Kablak-Ziembicka A. (2008). Carotid artery stenting with patient- and lesion-tailored selection of the neuroprotection system and stent type: early and 5-year results from a prospective academic registry of 535 consecutive procedures (TARGET-CAS). *Journal of Endovascular Therapy*.

[19] Randall M. S., McKevitt M. F., Kumar S. (2010). Long-term results of carotid artery stents to manage symptomatic carotid artery stenosis and factors that affect outcome. *Circulation. Cardiovascular Interventions*.

[20] Simonetti G., Gandini R., Versaci F. (2009). Carotid artery stenting: a single-Centre experience with up to 8 years' follow-up. *European Radiology*.

[21] Schofer J., Musiałek P., Bijuklic K. (2015). A prospective, multicenter study of a novel mesh-covered carotid stent: the CGuard CARENET trial (carotid embolic protection using MicroNet). *JACC. Cardiovascular Interventions*.

[22] Stabile E., de Donato G., Musialek P. (2020). Use of dual-layered stents for carotid artery angioplasty: 1-year results of a patient-based meta-analysis. *JACC. Cardiovascular Interventions*.

[23] Peters S. A., Huxley R. R., Woodward M. (2014). Diabetes as a risk factor for stroke in women compared with men: a systematic review and meta-analysis of 64 cohorts, including 775 385 individuals and 12 539 strokes. *The Lancet*.

[24] McGuire D. K., Zinman B., Inzucchi S. E. (2020). Effects of empagliflozin on first and recurrent clinical events in patients with type 2 diabetes and atherosclerotic cardiovascular disease: a secondary analysis of the EMPA-REG OUTCOME trial. *The Lancet Diabetes and Endocrinology*.

[25] Mazurek A., Borratynska A., Tomaszewski T. Long-term outcomes of the MicroNetcovered stent system routine use for carotid revascularization in stroke prevention: PARADIGM-extend 5 year evidence—ESC best poster.

[26] Cola C., Brugaletta S., Martín Yuste V., Campos B., Angiolillo D. J., Sabaté M. (2009). Diabetes mellitus: a prothrombotic state implications for outcomes after coronary revascularization. *Vascular Health and Risk Management*.

[27] Mazurek A., Partyka L., Trystula M. (2019). Highly-calcific carotid lesions endovascular management in symptomatic and increased-stroke-risk asymptomatic patients using the CGuard™ dual-layer carotid stent system: analysis from the PARADIGM study. *Catheterization and Cardiovascular Interventions*.

[28] Kumbhani D. J., Bavry A. A., Kamdar A. R., Helton T. J., Bhatt D. L. (2008). The effect of drug-eluting stents on intermediate angiographic and clinical outcomes in diabetic patients: insights from randomized clinical trials. *American Heart Journal*.

[29] Olesen K. K. W., Pareek M., Madsen M. (2020). Ten-year outcomes of sirolimus-eluting versus zotarolimus-eluting coronary stents in patients with versus without diabetes mellitus (SORT OUT III). *The American Journal of Cardiology*.

[30] Aikawa H., Kodama T., Nii K. (2008). Intraprocedural plaque protrusion resulting in cerebral embolism during carotid angioplasty with stenting. *Radiation Medicine*.

[31] Okazaki T., Sakamoto S., Shinagawa K. (2019). Detection of in-stent protrusion (ISP) by intravascular ultrasound during carotid stenting: usefulness of stent-in-stent placement for ISP. *European Radiology*.

[32] Shinozaki N., Ogata N., Ikari Y. (2014). Plaque protrusion detected by intravascular ultrasound during carotid artery stenting. *Journal of Stroke and Cerebrovascular Diseases*.

[33] Beckman J. A., Creager M. A., Libby P. (2002). Diabetes and atherosclerosis. *Journal of the American Medical Association*.

[34] Bonati L. H., Gregson J., Dobson J. (2018). Restenosis and risk of stroke after stenting or endarterectomy for symptomatic carotid stenosis in the International Carotid Stenting Study (ICSS): secondary analysis of a randomised trial. *Lancet Neurology*.

[35] Lal B. K., Beach K. W., Roubin G. S. (2012). Restenosis after carotid artery stenting and endarterectomy: a secondary analysis of CREST, a randomised controlled trial. *Lancet Neurology*.

[36] Brott T. G., Hobson R. W., Howard G. (2010). Stenting versus endarterectomy for treatment of carotid-artery stenosis. *The New England Journal of Medicine*.

[37] Tekieli L., Pieniazek P., Musialek P. (2012). Zotarolimus-eluting stent for the treatment of recurrent, severe carotid artery in-stent stenosis in the TARGET-CAS population. *Journal of Endovascular Therapy*.

[38] Tekieli Ł., Musiałek P., Kabłak-Ziembicka A. (2019). Severe, recurrent in-stent carotid restenosis: endovascular approach, risk factors. Results from a prospective academic registry of 2637 consecutive carotid artery stenting procedures (TARGET-CAS). *Advances in Interventional Cardiology*.

[39] Nerla R., Castriota F., Micari A. (2016). Carotid artery stenting with a new-generation double-mesh stent in three high-volume Italian centres: clinical results of a multidisciplinary approach. *EuroIntervention*.

[40] Nerla R., Micari A., Castriota F. (2018). Carotid artery stenting with a new-generation double-mesh stent in three high-volume Italian centres: 12-month follow-up results. *EuroIntervention*.

[41] Imamura H., Sakai N., Matsumoto Y. (2021). Clinical trial of carotid artery stenting using dual-layer CASPER stent for carotid endarterectomy in patients at high and normal risk in the Japanese population. *Journal of neurointerventional surgery*.

[42] Speziale F., Capoccia L., Sirignano P. (2018). Thirty-day results from prospective multi-specialty evaluation of carotid artery stenting using the CGuard MicroNet-covered Embolic Prevention System in real-world multicentre clinical practice: the IRON-guard study. *EuroIntervention*.

[43] Capoccia L., Sirignano P., Mansour W., Sbarigia E., Speziale F. (2018). Twelve-month results of the Italian registry on protected CAS with the mesh-covered CGuard stent: the IRON-Guard study. *EuroIntervention*.

[44] Sirignano P., Stabile E., Mansour W. (2021). 1-year results from a prospective experience on CAS using the CGuard stent system: the IRONGUARD 2 study. *JACC. Cardiovascular Interventions*.

[45] Narumi S., Sasaki M., Ohba H. (2013). Prediction of carotid plaque characteristics using non-gated MR imaging: correlation with endarterectomy specimens. *American Journal of Neuroradiology*.

[46] Kuwabara M., Sakamoto S., Okazaki T. (2022). Factors contributing to restenosis after carotid artery stenting and the usefulness of magnetic resonance plaque imaging: a study of 308 consecutive patients. *European Journal of Radiology*.

[47] Trystuła M., Musiałek P. (2020). Transient flow reversal combined with sustained embolic prevention in transcervical revascularization of symptomatic and highly-emboligenic carotid stenoses for optimized endovascular lumen reconstruction and improved peri- and post-procedural outcomes. *Advances in Interventional Cardiology*.

[48] Galyfos G. C., Tsoutsas I., Konstantopoulos T. (2021). Editor's choice - early and late outcomes after transcarotid revascularisation for internal carotid artery stenosis: a systematic review and meta-analysis. *European Journal of Vascular and Endovascular Surgery*.

